# Hospital News

**Published:** 1922-11

**Authors:** 


### HOSPITAL NEWS.

The Countess of Lonsdale has opened the extension to Penrith Cottage Hospital, which includes two new wards.

Mr. C. F. de Salis, chairman of the Middlesex County Council, has opened the Council's new sanatorium at Harefield Park. The house accommodates the staff, and a series of hut-wards the patients—men, women and children.

A well-known sporting paper has lately suggested that all who believe that " wild oats should be sown " ought to visit the wax models of diseased tissue in the pathological department of Guy's Hospital.

Mr. Roger Beck, the generous benefactor of Swansea Hospital, has been presented with his portrait, which he has handed to the hospital. The artist is Miss Gwenny Griffiths, whose father is a doctor in the town, and whose brother is one of the hospital's honorary surgeons.

A former controller of stores at the Ministry of Pensions Hospital, Orpington, has been sentenced to 12 months' hard labour for stealing provisions. The prisoner's previous record included the award of the Meritorious, Long Service and Good Conduct Medals, and his appointment as a Member of the Order of the British Empire.

The Swansea Hospital has received a letter from the Royal College of Physicians and Surgeons saying that the hospital cannot be recognised for more than six months' study unless attached to a recognised medical school. The hospital has explained its connection with the Welsh Medical School. It has already four pupils, and it is hoped that these will prove the beginning of a medical school.

The extension of the Lowestoft and North Suffolk Hospital, for which the money has been raised as a war memorial, has been criticised on the ground that a local tender was not accepted by the Building Committee. A statement by Colonel A. G. Lucas, the hospital's president, has given the reasons for this decision, and the agitation seems to be an example of the thoughtlessness with which hospitals are criticised, too often by those who do not otherwise offer much " support."

After undergoing four operations, a patient in the Royal Hampshire Hospital committed suicide by taking lysol in the ward lavatory. The coroner said that, as this was a poison, it should be kept locked up, but the house surgeon declared that it was used so frequently that to lock it up was impracticable ; also that it could be purchased anywhere without restriction. The coroner appreciated the difficulties, but stated that in his opinion something must be devised to prevent patients from having access to lysol. His views were brought before the house committee.

At a meeting in Edinburgh on Oct. 5, representative of registered nurses throughout the country, including members of the College of Nursing, the Scottish Matrons' Association, Queen's Nurses and others, a committee was formed to secure candidates, representative of every branch of the profession, for election to the General Nursing Council for Scotland. Membership of the Council involves a considerable amount of work, and frequent attendances at meetings in Edinburgh, and the committee has ascertained that the nurses chosen will be able to attend the meetings and take an active part in committee work. It has agreed to recommend the following candidates : Miss Gill, R.R.C. (Lady Superintendent of Nurses, Royal Infirmary, Edinburgh) ; Miss White (Superintendent, Queen Victoria's Jubilee Institute for Nurses, Scottish Branch, Edinburgh) ; Miss Merchant (Matron, Stobhill Hospital, Springburn, Glasgow) ; Miss Burleigh (Matron, Royal Hospital for Sick Children, Edinburgh) ; Miss Fraser, R.R.C. (Matron, Gray's Hospital, Elgin) ; Miss Edmondson, R.R.C. (Matron and Superintendent, Royal Infirmary, Aberdeen) ; Miss Davidson, R.R.C. (Matron, Mental Hospital, Bangour) ; Miss Thomas (Matron, City Fever Hospital, Edinburgh) ; and Miss Agnes Boyd Donald (Private Nurse, 18 Berkeley Terrace, Glasgow.)

A new orthopædic and electro-therapeutic department has been opened, with other improvements to the out-patient department, at Cheltenham Hospital.

Brancepeth Castle, near Durham, has been generously offered by Viscount Boyne to the Voluntary Hospital Committee of Durham County as an auxiliary hospital.

Princess Mary, accompanied by Viscount Lascelles, has opened the new infants' ward at Leeds General Infirmary. The ward was built by the citizens of Leeds as a wedding present to the Princess.

The Not-Forgotten Association, on behalf of still incapacitated service-men, has taken over Lonsdale House, Clapham Park, one of the smaller hospitals maintained by the Ministry of Pensions, which would otherwise have been closed. The hospital provides for 30 cases of spinal disability.

The Yorkshire branch of the British Dental Association, meeting at Leeds, witnessed in the dental section of the General Infirmary a demonstration of the gasotherme, a painless method of stopping teeth, for which a patient volunteered her services.

The Women's Freedom League complains that " even the hospitals exclusively for women and those for children have no women on their management committees," and that " appointments to the staff," made by these committees, offer " little chance " to women doctors.

The twentieth annual session was opened at the Missionary School of Medicine, 82 Wimpole Street. The course, intended to instruct missionaries in the elements of medicine, is carried on in connection with a general hospital. Four hundred students have passed through the school.

The Royal College of Physicians, Edinburgh, has awarded the Victoria Jubilee Cullen prize " for the greatest benefit to practical medicine," equally to Dr. John Thomson, consulting physician to the Royal Edinburgh Hospital for Sick Children, and to Dr. Norman Walker, University lecturer on Dermatology.

A useful list of scraps that may be converted into money has been published in connection with the Shipley (Yorks.) tinfoil collection on behalf of Sir Titus Salt's Hospital. The list includes silver paper from sweets and cigarettes, capsules from bottles, tooth paste or paint tubes, tobacco and tea lead, metal photograph frames. The scrap is smelted and sold by the Central Druids' Collection for Hospitals.

The Tyneside and Wearside Committees of the Industrial Orthopædic Society, which supports the Manor House Hospital, Hampstead, lately welcomed Dr. A. E. Woodall, the hospital's senior surgeon. The hon. treasurer, Mr. Asquith, and the Northern organiser, Capt. Parker, declared that the hospital must be treated as an extra by industrial workers, for they did not wish to take away a penny from other hospitals.

In connection with the recent gift day for the London hospitals, when some hundreds of tons of allotments produce were collected, the National Union of Allotment Holders has received many letters of thanks. In a few cases the smaller hospitals have been supplied with potatoes to last for several weeks. Guy's Hospital, with its 600 patients, received enough cabbages for three days, and potatoes for nearly a week.

The authorities of St. Thomas's Hospital have raised the question of the insurability of nurses before July 1, 1922, and the Minister of Labour has decided that before that date a senior staff nurse at St. Thomas's was insurable under the Unemployment Insurance Acts. Against this decision the authorities of St. Thomas's have lodged an appeal, the main ground of which is that the senior staff nurse is employed in domestic service. This appeal will probably be heard early next month.

